# ELViS: an R package for estimating copy number levels of viral genomic segments at base-resolution

**DOI:** 10.1093/bioinformatics/btaf622

**Published:** 2025-11-12

**Authors:** Jin Young Lee, Jeremiah R Holt, Xiaobei Zhao, Katherine A Hoadley, D Neil Hayes, Hyo Young Choi

**Affiliations:** UTHSC Center for Cancer Research, University of Tennessee Health Science Center, Memphis, TN, 38163, USA; UTHSC Center for Cancer Research, University of Tennessee Health Science Center, Memphis, TN, 38163, USA; UTHSC Center for Cancer Research, University of Tennessee Health Science Center, Memphis, TN, 38163, USA; Department of Genetics, Computational Medicine Program, Lineberger Comprehensive Cancer Center, University of North Carolina, Chapel Hill, NC, 27599, USA; UTHSC Center for Cancer Research, University of Tennessee Health Science Center, Memphis, TN, 38163, USA; UTHSC Center for Cancer Research, University of Tennessee Health Science Center, Memphis, TN, 38163, USA; Department of Preventive Medicine, Division of Biostatistics, University of Tennessee Health Science Center, Memphis, TN, 38163, USA

## Abstract

**Motivation:**

Tumor viruses account for ∼10% of cancer diagnoses. Virally induced tumorigenesis is understood as direct signaling through oncogenes such as E6 and E7 genes in the case of human papillomavirus. Furthermore, pathogen characteristics such as viral oncogene dose may impact the disease course. To our knowledge, no tool has been proposed to assess the intra-viral copy number alterations that define the gene dose of viral oncogenes and associated suppressive pathways native to the pathogen’s normal life cycle.

**Results:**

We propose an R package, “ELViS,” that analyzes viral copy number changes from DNA sequencing of whole viral genomes. The method adjusts for viral load with 2D transformation and segmentation to offer the relative viral gene doses.

**Availability and implementation:**

The ELViS R package is available from https://bioconductor.org/packages/ELViS. This article used controlled access data from dbGaP (phs001713.v1.p1).

## 1 Introduction

Approximately 10% of human cancers are attributable to infections of tumorigenic viruses (White *et al.* 2014) with the viral oncogenes inducing malignant transformation of host cells. Viral signaling is a delicate balance of cell growth and suppressive mechanisms that ensure the completion of the viral life cycle. Virally mediated tumorigenesis may occur as an unintended result. For instance, high-risk human papillomaviruses (HPV), which cause the highest proportion of viral cancers, encode E6 and E7 genes (hereafter referred to as E6/E7) promoting degradation of tumor suppressors p53 and pRB, whereas E4 partially antagonizes by completing the life cycle. Imbalance between E6/E7 and E4 may contribute to the malignant phenotypes, including resistance to apoptosis and accelerated cell proliferation (Pal and Kundu 2019). It has been known that aberrant viral genomes are generated via disrupted viral replication, often in the context of tumorigenesis. Consideration of the resulting altered genomes highlights the importance of characterizing viral gene dosage and genome structure in elucidating HPV-associated cancer pathology. Methods of assessing viral gene dose are currently lacking.

Existing approaches like VirusFinder (Wang *et al.* 2013) and VERSE (Wang *et al.* 2015) focused on viral integration into the human genome, potentially altering host cancer gene expression. Alternatively, DI-tector (Beauclair *et al.* 2018) and DVG-profiler (Bosma *et al.* 2019) find RNA viral structural diversity in non-cancerous diseases, focusing on splice junctions. No method, to our knowledge, interrogated the integrity of viral genomes or gene dosage changes caused by viral DNA structural alterations.

The lack of methods may in part be due to technologic factors. Viral integration detection by chimeric reads requires coverage far <100X, insufficient to characterize viral gene dosage. Recently, high-depth approaches such as whole viral genome capture provided an exquisite level of viral sequencing detail. Importantly, although similar in concept to host genome copy number changes, viral copy number alterations occur on a much smaller scale (aka “micro”), involving a “few” bases/hundreds of bases. Therefore, techniques applicable to large genomes (thousands to millions of bases) based on binned representations cannot be applied, necessitating single-base resolution (aka “base-level”).

To address this, we developed “ELViS (Estimating Copy Number Levels of Viral Genomic Segments),” an R package that analyzes micro copy number changes in DNA viral genomes. ELViS utilizes a shape property of base-level read depth, which can be conceptualized as a data object similar to the “pileup” file format (Li *et al.* 2009). A similar technique has been reported for the identification of abnormal transcripts in RNA (Choi *et al.* 2021). ELViS is publicly available at https://bioconductor.org/packages/ELViS.

## 2 ELViS workflow and outputs

**Figure 1.**
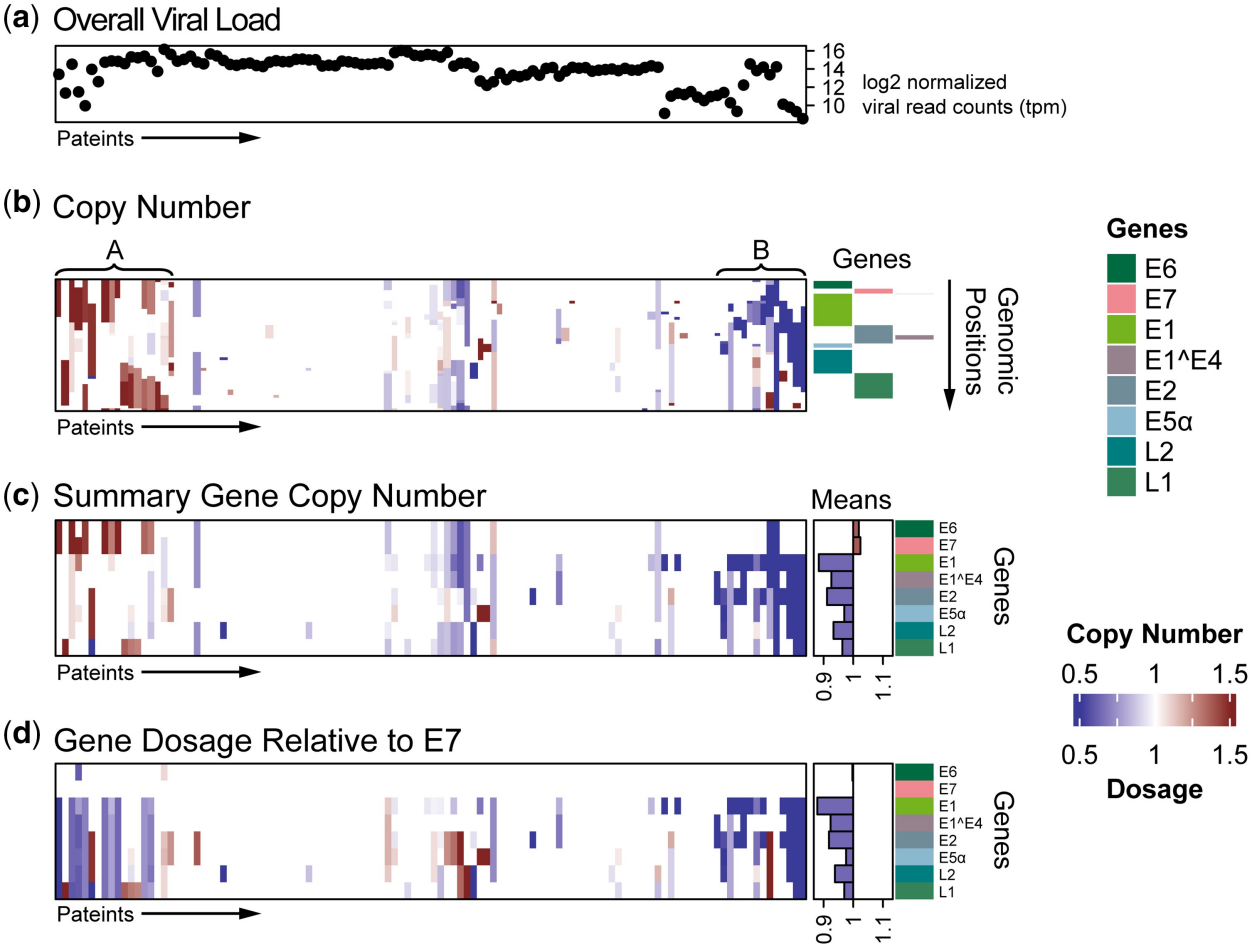
Heatmaps for viral copy number variations of 114 HNSCC patients. Columns in all panels indicate patients and are in the same order based on integrative clustering (Supplementary Methods). (a) Overall viral load measured by normalized viral read counts expressed as tags per million (tpm), on the *y*-axis in log2 scale. (b) Copy number calls. Rows are HPV16 genomic positions and colored bar panel on the right indicates positions of viral transcripts in HPV16 genome as indicated by color index. A and B indicate patient sample groups with duplications and deletions respectively. Panel c and d are heatmaps of (c) Gene-level copy number calls and (d) viral gene dosages relative to the E7 gene of HPV16. Rows are individual viral genes. To the right of the panel is shown the mean “across sample” copy number (c) or gene dosage (d) for each gene, indicating relative increase of the E6/E7 gene relative to other genes.

The ELViS workflow begins by generating base-level read depth profiles across any viral genome using BAM files. Viral copy number alterations manifest as abnormal shape changes of depth profile, while intact samples display consistent patterns (Fig. 1, available as supplementary data at *Bioinformatics* online). To reliably identify such alterations from uneven targeted sequencing data in a short viral genome, we developed a novel statistical framework incorporating 2D-transformation and 2D-segmentation. Only samples with sufficient depth are considered, as poorly covered samples exhibit unfavorable signal-to-noise properties.

*2D transformation.* The transformation step adjusts for position- and sample-specific variability caused by differences in enrichment efficiency and viral load. Raw read depth data, $X_{j}\in R^{d},\;j=1,\ldots ,n$, where *d* is viral genome length and *n* is the number of samples, are transformed into two complementary probabilistic domains: normalized read depths ($Y_{j}\in R^{d}$) and robust Z-scaled deviations ($Z_{j}\in R^{d}$). $Y_{j}$ corrects for viral load by setting the median of unaltered genomic regions (baseline) of $X_{j}$ to 1, enabling accurate cross-sample comparisons (Supplementary Methods). $Z_{j}$ captures position-specific structural deviations by computing robust Z-scores from all $Y_{j}$‘s, i.e. $Y=[Y_{1},Y_{2},\ldots ,Y_{n}]\in R^{d\times n}$, which facilitates the identification of altered regions.

*Copy number variant detection.* ELViS integrates $Y_{j}$ and $Z_{j}$, as $Y_{j}$ is confounded by uneven read depths, while $Z_{j}$ is susceptible to distortions from heterogeneous noise levels across the viral genome. To leverage the complementary properties, the combined data are analyzed using a 2D segmentation algorithm (Patin *et al.* 2020). Breakpoints are subsequently determined to define copy number variants. Segments with similar read depth levels are grouped together to enhance interpretability (Supplementary Methods).

## 3 Results

### 3.1 Viral copy number alterations in head and neck squamous cell carcinomas sequencing data

Head and neck squamous cell carcinoma (HNSCC) is one of the most common cancers, and HPV is a driver event in >60% of the oropharyngeal subsite (The_Cancer_Genome_Atlas_Network 2015). To demonstrate ELViS, we analyzed copy number alterations in the HPV16 genome using a high-depth targeted sequencing platform that captures whole virus and host cancer genes, derived from 370 patients with HNSCC treated on the clinical trial NCT01457196 (Montgomery *et al.* 2016). Of them, the ELViS algorithm was applied to 114 tumors determined to be HPV(+) with sufficient coverage for downstream analysis (Fig. 1 and Fig. 2, available as supplementary data at *Bioinformatics* online, and Supplementary Methods).

We observed that the viral read counts showed exponential variations of 196-fold across samples (Fig. 1a) and 26-fold within a sample. Nonetheless, the pattern was reproducible across samples, such as the coverage nadir (blue stripe across all samples) located at the UTR near the 5’ end of E5α viral gene (Fig. 3b, available as supplementary data at *Bioinformatics* online). Coverage was normalized using the “baseline-corrected approach” as described in the methods above (Fig. 3c, available as supplementary data at *Bioinformatics* online). Robust Z scaling further improved signal detection (Fig. 3d, available as supplementary data at *Bioinformatics* online), correcting noisy regions such as the viral poly-A tail, a region of high sample-to-sample sequence heterogeneity associated with a nadir of coverage in the capture platform. Integrative clustering of multiple data matrices as described in Supplementary Methods clearly illustrated patterns of recurrent viral alterations (Fig. 1b). Deep deletions were associated with a low viral load, and duplications with higher viral loads (Fig. 1a and b). Copy number alterations convey a biologic interpretation of gene dose, and ELViS produces sample-level views to investigate target genes (Fig. 4, available as supplementary data at *Bioinformatics* online). Furthermore, we re-classified alterations based on the impact on viral transcripts to obtain gene-level copy numbers, which showed recurrent gains of the E6/E7 and loss of the E1(Fig. 1c, patient groups A and B). An alternative “gene dosage” is displayed in which copy number segments were indexed to the viral oncogene E7. In this view, most samples with alterations showed concordance, with genes located 3’ of E7 exhibiting a lower gene dosage compared to the viral oncogenes E6 and E7 for both patient groups A and B (Fig. 1d). This suggests that loss of segments 3’ of E7 or gain of E6/E7 may have biologic equivalence.

### 3.2 Performance evaluation with simulated data

We generated a simulation dataset using w-WESSIM2 (Tanner *et al.* 2019) (Supplementary Methods). Briefly, we generated *in silico* baits based on a selected representative sample with copy-neutral HPV16 from the set of 114 HPV(+) cases (Fig. 5, available as supplementary data at *Bioinformatics* online). We simulated deletions and duplications from 50 bp to 2 kb in the sequencing data with depths ranging from 50X to 1000X. ELViS achieved a sensitivity >95% for detecting deletions ≥50 bp and duplications ≥ 500 bp that changed the copy number at least by half, relative to baseline at 100X coverage depths, which falls within the depths of coverage of the typical whole exome sequencing assay (>100X) for cancer genomic data (Fig. 6, available as supplementary data at *Bioinformatics* online).

## 4 Conclusion

ELViS is a novel tool for detecting micro copy number alterations in any viral genome with single-base resolution read depth. The procedure performs well in targeted sequencing data with non-uniform read depths and does not rely on binning procedures. By applying ELViS to capture sequencing dataset from the NCT01457196 clinical trial, we discovered copy number changes in the HPV16 genome usually occur in a way that they elevate the relative dosages of viral oncogenes, with relevance to the biology of head and neck carcinomas and HPV. Furthermore, considering that HPV(+) HNSCC patients with low viral load have been associated with poor prognosis in multiple studies (Mellin *et al.* 2002, Veitía *et al.* 2020, Hashida *et al.* 2021), examining the load of viral genes using ELViS in finer resolution will help define attractive categories of virus-associated cancer patients with clinical significance.

## Supplementary Material

btaf622_Supplementary_Data

## Data Availability

Targeted sequencing data used in this study are available in dbGaP under accession number phs001713.v1.p1, generated as part of clinical trial NCT01457196.
