# Supplementary material for: ELViS: an R package for estimating copy number levels of viral genomic segments at base-resolution
Source: Bioinformatics. 2025 Nov 12;41(12):btaf622. doi: 10.1093/bioinformatics/btaf622 (PMC12684729; doi:10.1093/bioinformatics/btaf622)
Supplement: btaf622_Supplementary_Data [file btaf622_supplementary_data.docx]

Supplementary Materials

ELViS: An R Package for Estimating Copy Number Levels of Viral Genomic Segments at Base-Resolution

Jin Young Lee^1^, Jeremiah R. Holt^1^, Xiaobei Zhao^1^, Katherine A. Hoadley^2^, D. Neil Hayes^1†^
, Hyo Young Choi^1,3†^

^1^UTHSC Center for Cancer Research, University of Tennessee Health Science Center, Memphis, TN

^2^Department of Genetics, Computational Medicine Program, Lineberger Comprehensive Cancer Center, University of North Carolina, Chapel Hill, NC

^3^Department of Preventive Medicine, Division of Biostatistics, University of Tennessee Health Science Center, Memphis, TN

^†^Corresponding Authors

# Supplementary Methods

## Generation and normalization of single-base resolution depth profile from read alignment files.

Single-base resolution depth profiles were generated from BAM alignment files of all samples using RSamtools. To exclude false discovery in HPV(−) patients or poorly covered samples, samples were excluded if the maximum depth of coverage over the HPV genome of interest was less than the low-depth threshold determined after examining the distribution over the entire dataset (Figure S2). We next applied upper quartile (Q3) normalization to obtain a uniform data range. Single-base resolution depth profiles were partitioned into windows when consecutive read depths transitioned across Q3. The mean of the normalized depths was obtained for each window and scaled using the robust scaling method. To account for the skewness of the data, median absolute deviations (MADs) were calculated separately for the values above and below the median. Median normalization was then applied, where the median was calculated excluding outlying segments with absolute values of scaled mean depth higher than the default cutoff of 4.

## Viral genome segmentation and baseline-correction normalization.

The primary purpose of our methodology was to report copy number variations in the viral genome from targeted sequencing data. Read depths in targeted sequencing data could not be directly compared between different genomic positions due to their distinct distributions, mainly due to differential enrichment efficiency. To accommodate this behavior, we applied a 2D segmentation approach (Patin et al., 2020) to the normalized read depth data and the robust-scaled deviations. After determining the optimal segmentation, the overall depth profiles were recentered by setting the mean of the segment least deviating from the median as a new baseline, which we define as baseline-correction normalization process. The recentering procedures were run once again with the updated normalized depth profile to obtain stable and converged results. For samples not feasible for 2D segmentation due to extended non-covered regions, 1D segmentation was applied to the scaled data to obtain segments.

## Base-resolution breakpoint detection.

Breakpoints were obtained by examining the transition in depth between adjacent positions. Positions were selected as breakpoints if the depth differences were more than 10 times higher than the 95^th^ percentile among the positions within 250bp. Those more than 5 times higher were also selected if the differences exceeded 8% of the reference level. Those with no more than 10 raw depth differences were filtered out to remove random transitions within the noise range. After dividing the intervals using these breakpoints, the median depth of the non-outlying interval (max Z-score < 30) with the lowest Q1 (lower quartile) was set as a new reference level to obtain a refined depth profile. The process was iterated 2 times for the refined depths to achieve stable and converged results.

## Grouping regions with similar read depth levels.

Segments were considered as baseline if their mean depths were within 1 SD of the depths in baseline segments for both normalized read depth $Y_{j}$ and robust z-scores $Z_{j}$. The remaining non-baseline segments, which were likely to contain copy number events, were categorized as gain segments if their mean depths were greater than that of the baseline segments, or as loss segments otherwise. To group similar gain or loss segments, similarity networks were constructed, with nodes indicating segments and edges indicating the proximity between them. Edges were created if the mean depths of the segments represented by the associated nodes were within 1 pooled SDs for both normalized read depth $Y_{j}$ and robust z-scores $Z_{j}$. Scaled absolute distances between nodes, $SAD_{mn}$, where m and n were different nodes, were defined as absolute mean difference scaled by pooled SD. Edge weights were defined as similarity metrics calculated as $\frac{1}{SAD_{mn}+1}$. Fully connected subnetworks that did not overlap with each other were selected, starting from those with the largest sum of edge weights. Segments in a selected subnetwork were treated as having the same copy number level.

## Integrative clustering of multiple data matrices.

The overall viral dosage and viral gene dosages exhibited dynamic ranges, which necessitated the examination of raw read depth. Read depth reflected overall viral dosage and normalized read depth emphasized relative dosage differences between viral genes. In order to address both absolute and relative properties, a simple and fast clustering algorithm was employed to integrate distance measures from multiple data matrices. For each data matrix $X_{i}$, we obtained a pairwise distance matrix $D_{i}$. Then each $D_{i}$ was normalized by dividing it by its $p$-th percentile. $p$ was set to the upper quartile (Q3) by default. To prevent the case where Q3 was zero, the percentile of the mean distance multiplied by 1.1 was used in place of Q3 if it was higher than Q3. If any of the distance matrices showed $p>0.75$, $p$ was updated to the maximum value of all the distance matrices. Normalized distance matrices, ${ND}_{i}=D_{i}/(p-th percentile)$, were then averaged to obtain an integrative distance matrix ($ID$). $ID$ was then subject to hierarchical clustering with Euclidean distance and complete linkage method. Sample orders in Figure 1 and Figure S3 were determined by this procedure. The heatmaps were drawn using ComplexHeatmap R package (Gu, 2022).

## Gene copy number and relative gene dosage

In samples with complex copy number profiles where a variant did not entirely cover a gene, the interpretation of gene dose was considered challenging. For simplicity, we chose to quantify the number of intact gene copies to potentially capture a concept of functional genes. The gene copy numbers used in Figure 1c were obtained by taking the minimum copy number among the overlapping segments. Furthermore, relative dosages between genes could be of interest, when the balances between genes might represent critical biologic function. For this, we defined relative gene dosages by dividing functional gene copy numbers of all genes by that of the gene of interest. These metrics were used in Figure 1d.

## ELViS output tables and figures

ELViS provides the coordinates of detected variants and their copy number levels, along with summary statistics of raw read depths ($X_{j}$), normalized read depths ($Y_{j}$), and robust Z-scaled deviations ($Z_{j}$). The copy number calls and different layers of data, $X_{j}$, $Y_{j}$, and $Z_{j}$ are visualized together as base-level read depth line plots and as heatmaps. For intuitive observation of the associations of viral loads and viral gene dosage with tumor biology on heatmaps, ELViS provides integrative clustering of multiple matrices ($X$, $Y$, $Z$, and copy number levels). Gene-level copy number estimates are generated relative to the baseline. Alternatively, ELViS offers the option to index copy numbers to a user-selected gene or segment.

## Panel sequencing data simulation for performance evaluation

To evaluate the performance of ELViS in detecting true variants, we generated simulation data as proposed by w-WESSIM2 (Tanner et al., 2019). First, we selected a representative sample from the set of 114 HPV(+) cases which was identified in the analysis as having no copy number changes but which was otherwise deeply covered and generated the in-silico baits required to simulate sequencing libraries (Figure S5). We simulated variants by duplicating and deleting sequences of 50bp, 500bp, 1Kbp, and 2Kbp in the HPV16 reference genome (gi|333031|lcl|HPV16REF.1) for duplications and deletions, respectively. Systematic errors resembling Illumina sequencing data of human samples generated from NovaSeq 6000(Hs-Nova-TruSeq.reseq) were introduced to FASTA files with and without variants using ReSeq (Schmeing and Robinson, 2021).

*reseq illuminaPE -r Simulated_noNs.fasta -s Hs-Nova-TruSeq.reseq --stopAfterEstimation --writeSysError Simulated_noNs_syserrors.fq*

Next, sequencing reads aligned to the HPV16 reference genome were extracted and used as in-silico bait to reproduce the realistic read depth profile of the original data. Extracted reads were aligned to FASTA files with an error profile generated in the earlier step using pblat (Wang and Kong, 2019) to get psl alignment files.

*pblat Simulated_noNs.fasta.2bit HPV16_FASTA.fa Bait_alignment.psl -threads=10 -minScore=70 -minIdentity=95*

The psl files were used as probability distributions from which to generate baited sequencing libraries (*Simulated_fragments.fa*) using w-WESSIM2. Insert size and standard deviations were set to the values of the original sample.

*python2 w-wessim2.py -R Simulated_noNs.fasta -S Simulated_noNs_syserrors.fq -B Bait_alignment.psl -N (Number of target read pairs) -O Simulated_fragments.fa -T Hs-Nova-TruSeq.reseq -m 20 -f 200 -d 70*

Sequencing reads were extracted from the baited sequencing libraries using *reseq seqToIllumina*. Only 5’ 76 base pairs of the reads were kept to make it resemble the original data with the same read lengths.

*reseq seqToIllumina -s Hs-Nova-TruSeq.reseq -i Simulated_fragments.fa -o Simulated_reads.fq*

The resulting simulated reads were aligned to the HPV16 reference genome with bwa mem (Li and Durbin, 2010). Aligned reads of samples with and without simulated variants were mixed in appropriate proportion to produce samples with different extents of copy number events: -90% and -50% for deletions and +50%, +100%, +200%, and +400% for duplications. For instance, a simulated BAM file with a deletion and the one without it were mixed in a 9:1 proportion to produce a copy number loss of -90%. ELViS was applied to the mixed BAM files to get copy number events and performance metrics: sensitivity, specificity, negative predictive values (NPV), positive predictive values (PPV), Accuracy, and F1 score (Figure S6).

Supplementary Figures


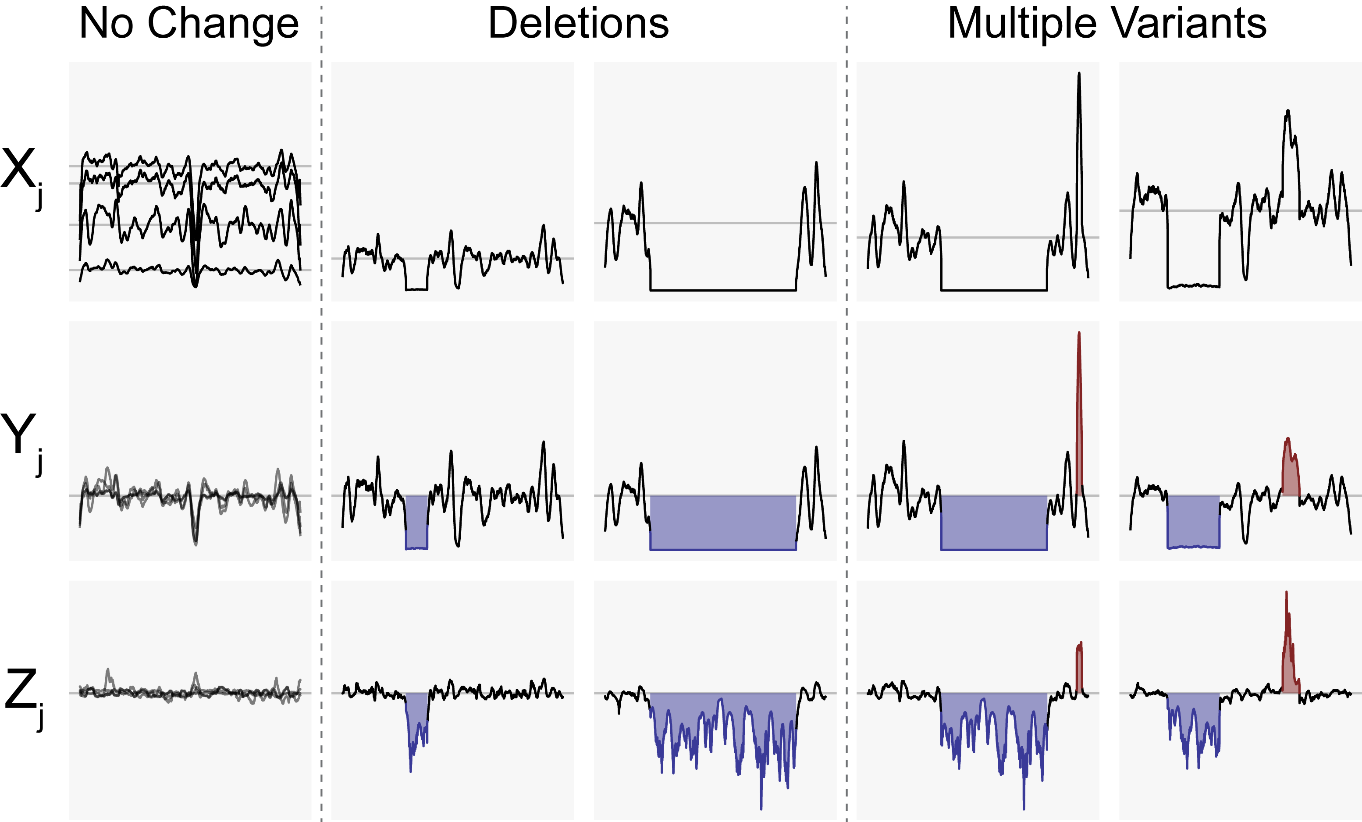


**Figure S1. Shape properties of base-resolution read depths from enrichment sequencing data.** Shapes of base-resolution read depths in samples with no copy number changes (left), deletions (middle), or multiple copy number variants (right). X-axes indicate viral genomic positions. On the y-axes, $X_{j}$ is raw read depth, $Y_{j}$ is baseline-corrected read depth, and $Z_{j}$ is robust scaled deviation sequence. Gray horizontal lines indicate the baselines. Shaded blue areas indicate areas of deletions, and shaded red areas indicate areas of amplification.


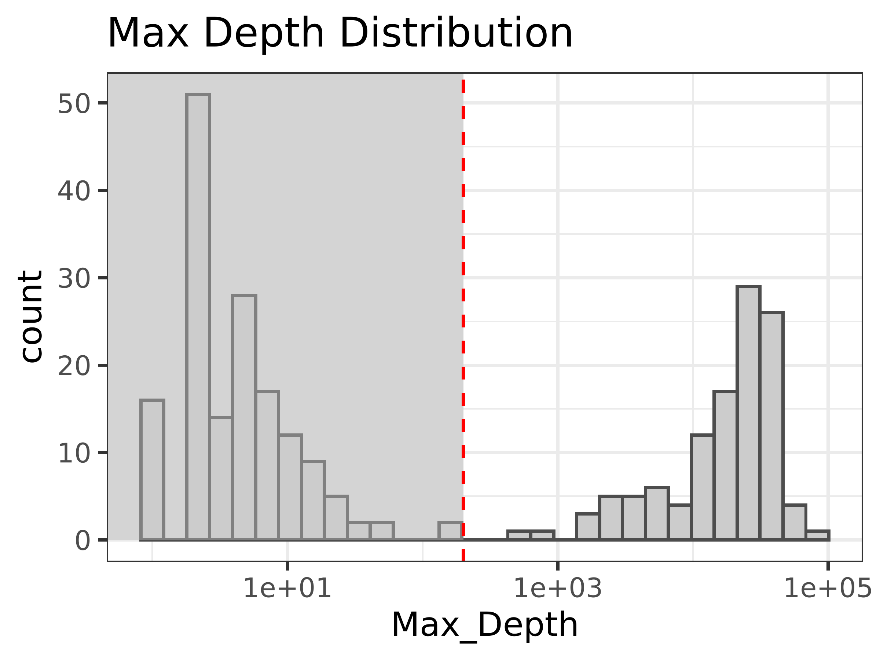


**Figure S2. Distribution of maximum read depth over the HPV16 genome.** The red dotted line indicates the filtering threshold, which is set to 200 in this case. The gray area indicates the samples that fall below the filtering threshold.


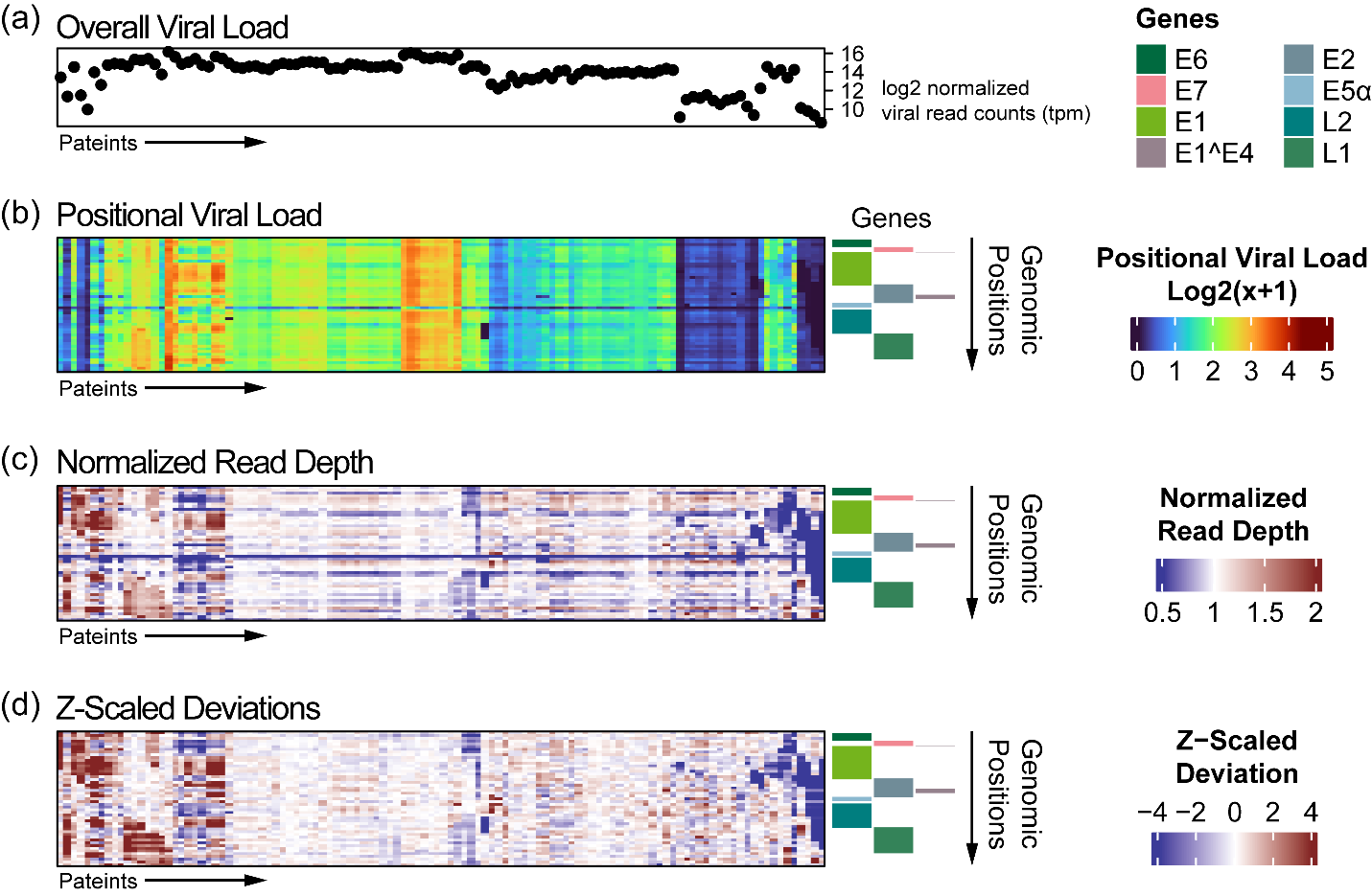


**Figure S3. Heatmaps of intermediate data for viral copy number analysis of 114 HNSCC patients.** Columns in all panels indicate patients and are in the same order based on integrative clustering (Supplementary Methods). (a) Overall viral load measured by normalized viral read counts expressed as tags per million (tpm), on the y-axis in log2 scale. Panel b-d are heatmaps showing in each row per-base metrics of (b) Positional viral load defined by read depth scaled by total aligned read depth, (c) normalized read depth, and (d) robust Z-scaled data over HPV16 genome. In panels b-d, Rows are HPV16 genomic positions and colored bar panel on the right indicates positions of viral transcripts in HPV16 genome as indicated by color index.


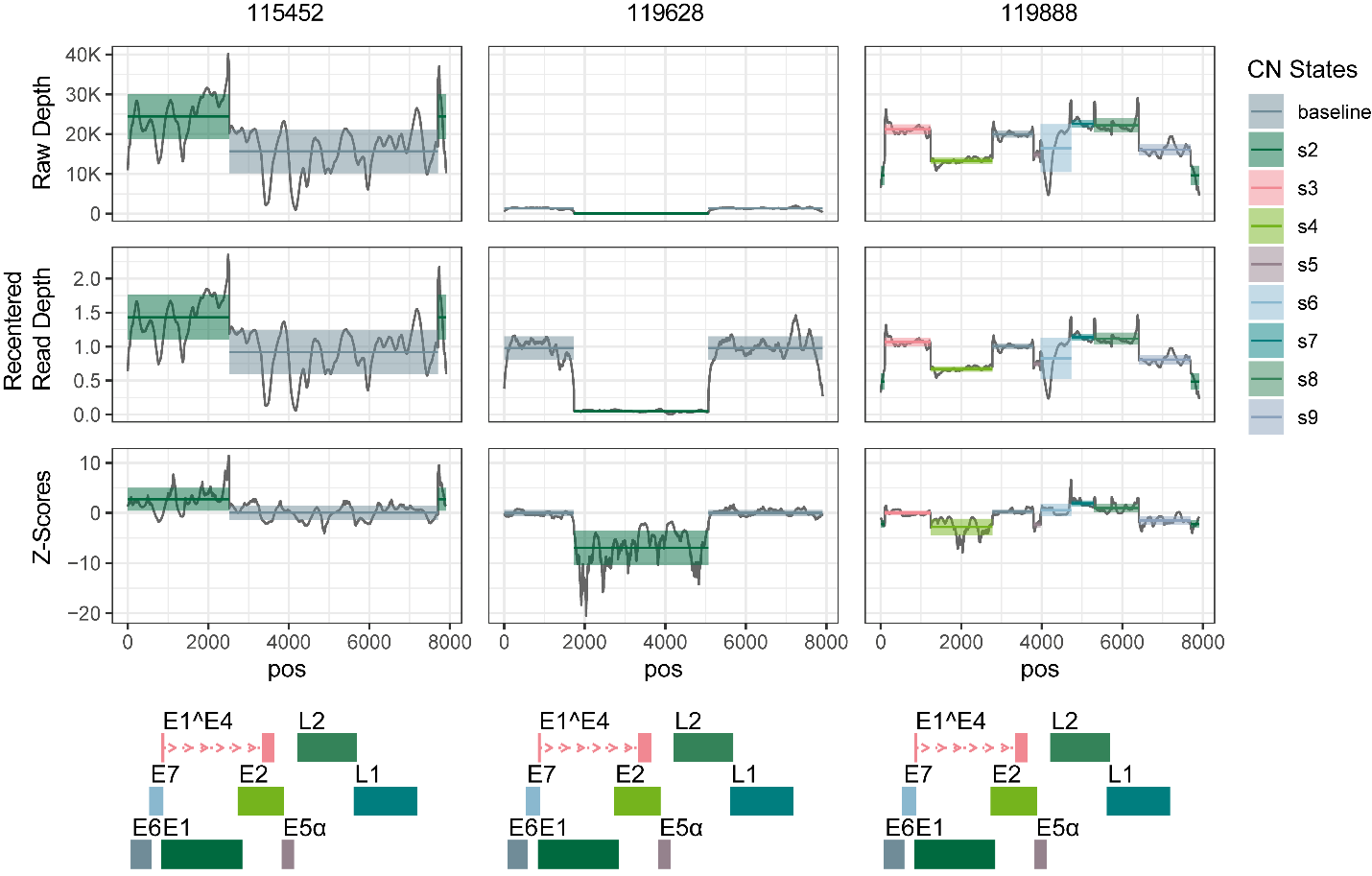


**Figure S4. Read depth profiles of example samples with relatively higher copy numbers of E6 and E7 and lower copy numbers of E1 and E2.** The numbers at the top indicate sample IDs. Colored transparent boxes indicate different copy number states designated by ELViS. Panels at the bottom indicate the areas that viral genes occupy.


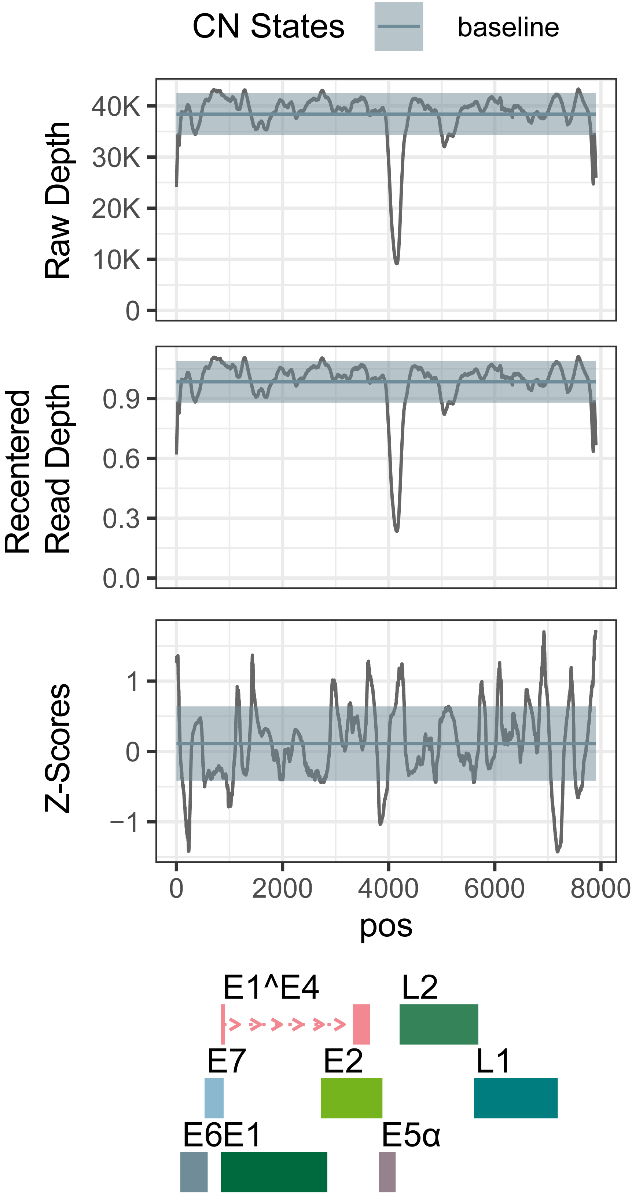


**Figure S5. Read depth profile of the sample (110008) used as *in-silico* baits for the simulation.** Colored transparent boxes indicate different copy number states designated by ELViS. Panels at the bottom indicate the area that viral genes are occupying.


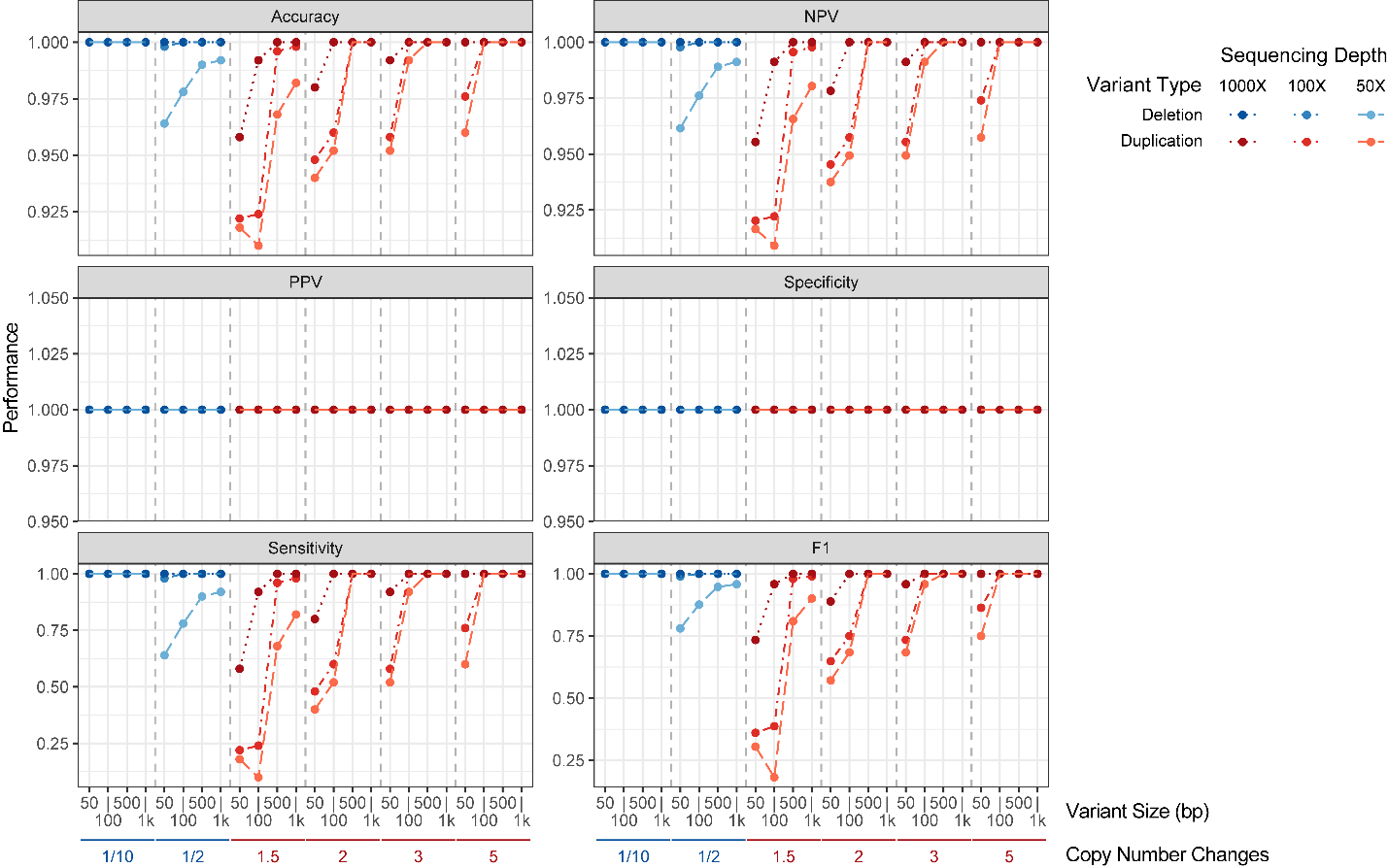


**Figure S6. Performance on simulated panel sequencing data.** Performance of ELViS according to multiple conditions of sequencing depths, variant size, variant types, and extent of copy number changes. Copy number changes represent the fold changes relative to the baseline. Blue and red dotted lines indicate metrics for deletions and duplications, respectively. Each panel shows each of the performance metrics: Accuracy, NPV, PPV, Specificity, Sensitivity, and F1 Score.

# Reference

Gu, Z. Complex heatmap visualization. *Imeta* 2022;1(3):e43.

Li, H. and Durbin, R. Fast and accurate long-read alignment with Burrows-Wheeler transform. *Bioinformatics* 2010;26(5):589-595.

Schmeing, S. and Robinson, M.D. ReSeq simulates realistic Illumina high-throughput sequencing data. *Genome Biol* 2021;22(1):67.

Tanner, G.*, et al.* Simulation of heterogeneous tumour genomes with HeteroGenesis and in silico whole exome sequencing. *Bioinformatics* 2019;35(16):2850-2852.

Wang, M. and Kong, L. pblat: a multithread blat algorithm speeding up aligning sequences to genomes. *BMC Bioinformatics* 2019;20(1):28.
